# Rabies and Hydrophobia1Read at the Thirty-seventh Annual Meeting of the American Veterinary Medical Association, Detroit, September, 1900.

**Published:** 1900-11

**Authors:** D. E. Salmon

**Affiliations:** Chief of Bureau of Animal Industry


### RABIES AND HYDROPHOBIA.[1]

By D. E. Salmon, D.V.M.,
CHIEF OF BUREAU OF ANIMAL INDUSTRY.

(Concluded from page 608.)

*The Diagnosis of Rabies.* The question has been raised as to whether the tests by the inoculation of rabbits and guinea-pigs on the surface of the brain with emulsions of the medullas of suspected animals is a reliable indication of the existence of rabies. The editor of one of our veterinary journals says: "Is it not about time to give this subject a rest until more real scientific work is done in this field of research, and some more accurate work completed beyond those tests on rabbits and guinea-pigs, so lacking in conclusiveness to a majority of scientific minds?"

It certainly must be well known to the writer of that editorial that any doubts as to the reliability of the test by inoculating rabbits and guinea-pigs may be very easily dispelled by returning to the old method of inoculating large animals in the skin or muscular tissue with the saliva or with the brain or spinal cord of affected individuals. The experiments recorded by Zinke, Count Salm, Berndt, Hertwig, and Rey, which are quoted above, show with what certainty the saliva of rabid dogs produces rabies in dogs and sheep. These experiments have been repeated and confirmed by numerous investigators in recent years. It is not necessary to use emulsified medullas to obtain positive results; it is not necessary to inoculate intracranially; it is not necessary to use rabbits and guinea-pigs. This method has simply been adopted for ordinary use because of its convenience and because it produces disease in a maximum number of subjects after a minimum period of in-

[1] Read at the Thirty-seventh Annual Meeting of the American Veterinary Medical Association, Detroit, September, 1900.

cubation. The reliability of the method has been abundantly proved, and the results correspond both with the post-mortem indications and with inoculation of larger animals in the skin and muscles. Those who habitually use small animals, inoculating subdurally with medulla emulsion as a test, and who have from time to time confirmed their results by inoculating larger animals by various processes, have confidence in this method. Would it not be well for those who lack experience to arrange to have some comparative trials of different methods made before they discredit modern science and modern laboratories by publicly expressed doubts? Moreover, the facts do not warrant the statement that the method of diagnosis in general use is lacking in conclusiveness to a majority of scientific minds.

Permit me to relate briefly a typical case of rabies which occurred in the District of Columbia. A dog broke the chain with which he had been fastened and escaped from its owner's premises in Georgetown about 11 A.M., April 27, 1900. It ran for about two hours, covering six or eight miles. During this time it attacked fourteen persons and succeeded in biting two. It bit two horses and four dogs and attempted to bite several other animals, and was finally killed by a man whose brother it was viciously attacking. The post-mortem examination of this dog indicated rabies. Inoculated rabbits died of rabies. One of the horses, which was bitten in the nose, was taken to the experiment station of the Bureau of Animal Industry for safe-keeping. This horse developed rabies June 4th, after an incubation of thirty-eight days. The disease was very intense, the animal violently kicking, pawing, and tearing its flesh with its teeth. It died June 5th. Two sheep were inoculated with an emulsion of the medulla of this horse. With one the virus was injected at the base of the ear; with the other it was injected into the connective tissue behind the right shoulder. The former sheep remained well; the latter showed symptoms of rabies July 1st, after an incubation of twenty-six days. The first day of its illness this sheep was very vicious, attacking everything that came into its stall; if sticks were held toward it it would strike them with its head so violently as to knock them from the hands of a strong man; it also snapped and bit at sticks very much as would a vicious dog. The second day it was down on its side, paralyzed and unable to get up. It died July 2d.

If this disease, which developed in a dog, which was communicated by biting to a horse, and which was inoculated by virus taken from the medulla upon rabbits and a sheep, was not rabies, what was it? Will those who express doubts as to the existence of rabies kindly inform us where in the veterinary literature of the world a disease other than rabies has been described which includes the features shown by these animals—a disease starting with the dog, communicable by biting and by inoculation with the fresh medulla, having twenty-six to thirty-eight days' incubation, characterized by excitability, viciousness, and paralysis, and ending in death in the course of one or two days? The speaker can only refer these characters to one disease, and that disease is rabies. The nature of the disease was clearly indicated by the post-mortem examination of the dog and by the inoculation of rabbits. It was unquestionably the same disease as affected the other dogs tested in the laboratory and to which reference has already been made.

The assertion made by a few individuals that an identical disease may be produced in experimental animals by inoculating subdurally with brain-substance from healthy animals or even with inert substances is not supported by facts. That paralysis and death may be caused by simple injuries to the brain is evident; but the animals so injured do not show the long period of incubation of rabies; they do not present the symptoms of nervousness, restlessness, irritability, viciousness, fury, and paralysis in succession, and leading to death in a few days after the appearance of the first symptoms; they do not develop virulent saliva by which the disease may be communicated from animal to animal by means of subcutaneous or intramuscular inoculations. That is, rabies is a contagious disease, the most prominent characteristic of which is the virulence of the saliva; and paralysis produced by mechanical injuries to the brain can no more be accepted by pathologists as rabies than can ulcers in the nose produced by caustics be accepted as glanders, or than inflammation of the lungs produced by the injection of oil of turpentine can be accepted as contagious pleuro-pneumonia.

*Rabies in Man.* Admitting the existence of rabies in dogs and other animals, Is the disease communicable to man? Are we to accept the statement so frequently made that all of the alleged cases of rabies in man are nothing more than a mere nervous affection, developed as the result of worry and fear—the so-called lyssophobia? The men who make such a statement must either be very ignorant of comparative pathology or they must be very reckless in their assertions. Let some person who holds these views tell us of a single disease which is communicable between so many species and such unrelated animals as the horse, the ox, the sheep, the pig, the dog, the rabbit, and the guinea-pig, and which is not at the same time communicable to man. One of the commonest diseases affecting unrelated species of animals is tuberculosis. It is also the commonest disease of man. Another disease affecting all warm-blooded animals is anthrax. It is easily communicable to man. Glanders, a disease of the equine genus, may be communicated to dogs, cats, and other carnivora, and to guinea-pigs, and with difficulty to sheep, goats, and pigs; it is not communicable to cattle, yet it readily affects man. Actinomycosis affects cattle, swine, horses, and some other animals, and to this disease mankind is also susceptible. Cowpox affects horses and cattle, and is easily transmitted to man. Aphthous fever, though more particularly a disease of ruminants, affects also horses, dogs, cats, pigs, and poultry; it is frequently communicated to man. Tetanus affects widely separated kinds of animals and attacks mankind. Malignant œdema is a disease of various species of animals, and also of man. This covers the communicable diseases other than hydrophobia which affect widely separated species of animals. The rule in comparative pathology, therefore, is that a disease which may be communicated to unrelated species of animals may also be communicated to mankind.

It is easily shown by inoculation experiments, which may be repeated at any time, that rabies affects as wide a range of subjects in the animal kingdom as do any of these diseases; it is not confined to domesticated creatures, but develops in wild animals, birds, and monkeys. All analogy, therefore, would lead us to conclude that mankind should be susceptible to it. Add to this analogy the observation of thousands of instances of supposed transmission, covering a period of more than two thousand years, and the case becomes so strong that it requires much more than a mere assertion to disprove it.

We have, however, more evidence than this to prove that hydrophobia in man is not a mere mimetic disease. In the great debate on the subject of rabies which took place in the Paris Academy of Medicine in 1863, M. Tardieu,[1] referring to the official statistics of France, said: "We owe to them the ruin of a serious prejudice perpetuated by the obstinacy with which certain physicians have held to the non-existence of the rabies virus, offering themselves, with a courage worthy of a better employment, for an experiment in which they would be the first victims. Rabies, which for them

[1] Tardieu. Discussion sur la Rage. Bul. de l'Acad. de Méd., 1863, p. 1152.

is but a convulsive nervous affection originating most often from fear, the statistics show us developing at an age when the moral emotions are not yet born, when the imagination does not remain stricken with chimerical terrors—in early infancy. More than 30 cases of rabies have been noted in children under five years, some of them of two and three years." Since that time the communication of the disease to young children, some of them less than one year old, has frequently been recorded; but no one has explained how this could have occurred if the disease was simply due to the imagination.

The proof of rabies in mankind which appeals most strongly to the scientific man of to-day is the experimental demonstration. The comparative pathologist has always accepted such a demonstration as incontestable with other diseases. Is there any reason why it should not be equally convincing with rabies? When we have questioned the diagnosis of glanders we have inoculated a well horse from the suspected one, and if glanders developed in the inoculated animal we have concluded that the horse from which the inoculation was made must have had glanders. A disease cannot be communicated by inoculation if the individual from which the inoculation is made does not have it. Inoculation has, consequently, become the supreme test of anthrax, pleuro-pneumonia, foot-and-mouth disease, tuberculosis, Texas fever, and other contagious diseases. Now, what does the experimental method show about rabies in man?

Magendie[1] says: "We have (M. Dupuytren and I) injected in the radial vein of a young man affected with rabies about eight grains of gummy extract of opium, without any apparent result. I inoculated, with my confrère, Breschet, the saliva of this patient upon a dog, placing it under the skin of the head (*sous la peau du front*), and the animal became rabid at the end of about a month. Two dogs which were bitten by this one also became rabid after forty days. These last bit several other dogs, but without any bad result. In this series of experiments rabies was arrested, then, of itself at the third generation."

Earle, Hertwig, and Renault made similar inoculations from affected persons to rabbits, and obtained identical results. In recent years, since the subdural inoculation of rabbits has been a recognized method of diagnosis, there have been many inoculations of these animals with virus from the human subject which have

[1] F. Magendie. Journal de Physiologie Expérimentale, 1821, vol. i., p. 42.

caused the disease. There have been two cases in Washington in which the diagnosis of rabies was confirmed in this manner; and Dr. Law, as reported elsewhere, has inoculated both dogs and rabbits with human virus and produced the disease.

How can this evidence be set aside? How can such arguments be answered? They are not answered except by blatant assertions unsupported by facts. When the saliva of a man produces rabies in the dog upon which it is inoculated it is certain that the man had rabies. There is no other conclusion admissible. The virus of a contagious disease cannot be originated *de novo* as the result of anxiety and fear.

We must conclude, therefore, that rabies is communicable to man, and that the evidence of this is overwhelming and unimpeachable.

*The Prevention of Rabies.* Admitting that there is such a disease as rabies, admitting its frequency, admitting that it is communicable to man, what measures are indicated for its repression and eradication? The measures generally adopted have for their object the reduction of the canine population rather than any direct influence for the destruction of the virus or the prevention of contagion. Such measures consist in the levying of a tax upon dogs, with a requirement that the dogs wear a numbered tag to show that the tax has been paid. To prevent violations of this regulation it is usual to have a dog-catching force, whose duty it is to seize all dogs found in public places without tags, and if these are not redeemed within a specified time to destroy them.

The effect of such a regulation depends very much upon the amount of the tax and the energy and thoroughness with which the measure is enforced. Usually the requirements of this law or ordinance are evaded by a large number of dog-owners, and it is common to see on the streets dogs without tags. There is seldom an adequate force of dog-catchers, and the sympathies of many communities appear to be with those who violate the law rather than with those who endeavor to enforce it.

When there is an unusual prevalence of rabies among dogs, or when, unfortunately, some person contracts the disease, particularly if that person happens to be well known or prominent in the community, there may be a temporary exhibition of strict and energetic enforcement of the regulations; but as soon as the public alarm subsides the efforts are relaxed, the dog-catcher disappears, the dogs are seen upon the streets with or without tags, and all things go on as before the panic occurred. While the number of dogs is thus periodically reduced somewhat, it is seldom that this reduction is sufficient to have much effect upon the propagation of the disease. It is probable that the tendency at such times to keep dogs confined in order to prevent them from being seized has much more influence in arresting the propagation of rabies than has the mere reduction in numbers.

In nearly all cases these indirect measures have been proven insufficient and unsatisfactory. What other disease would we attempt to stamp out by simply killing off one-fourth or one-third of the animals of the species affected? And if this measure is not efficient with other contagious diseases, why should we expect it to be with rabies? It appears self-evident from a sanitary point of view that there should be some direct measures instituted to prevent the propagation of the contagion. Such a measure would be the quarantine and confinement of all dogs for a sufficient time to cover the ordinary incubation period of rabies. As the enforced and continuous confinement of dogs without open-air exercise for a prolonged period is detrimental to the animals, they may be allowed in public places under such conditions as will absolutely prevent them from biting—that is, the animals should wear an efficient muzzle, or they should be muzzled and led in leash. As rabies is only propagated in nature by biting, such a regulation if thoroughly enforced would at once stop the transmission of the disease and soon lead to its disappearance.

When this measure is inaugurated, however, it is at once opposed by a large class of citizens who hold it to be cruel and unnecessary. Some muzzles are unquestionably cruel, but a properly made muzzle is not cruel, nor does it greatly inconvenience the dog after he becomes accustomed to it. The authorities should, therefore, prescribe the kind of muzzle to be used, and should select one which covers the mouth with a wire cage, so as to prevent biting, but without interfering with the movements of the mouth and the ingestion of liquids.

There have been many who have denied the utility of the muzzle, the strongest argument being that dogs do not wear the muzzle at home, and when they develop rabies and escape it is always when they are unmuzzled. Admitting the force of this argument, it is, nevertheless, a fact that if all dogs were required to be muzzled when in public places the appearance of a dog without a muzzle would at once attract attention, leading persons to avoid it and causing its early seizure by the authorities. Children might be instructed that an unmuzzled dog was dangerous, and that they should keep at a distance from it, and especially that they should never touch or fondle such an animal.

The results which have been obtained by muzzling justify its enforcement wherever there is an outbreak of rabies. Most of us have heard of the experience of Berlin with this measure about the middle of the century. From 1845 to 1853, 278 rabid animals were received at the Berlin Veterinary School. This is an average of 35 a year. From March, 1852, to the same month in 1853, the number was 82, and from March, 1853, to the end of July there were 37 more. On July 20th it was ordered that the use of the muzzle should become general. From July to the close of the year but 6 cases were admitted. Only 4 cases were observed in the whole city during 1854, and but a single case in 1855. For the seven years following there was not a single case recorded.[1]

While some have attributed the disappearance of rabies from Berlin at the time mentioned to other causes, muzzling has been adopted in Germany as the principal reliance in repressing this disease. Consul-General Mason reports from Berlin to the State Department that "in Berlin, Frankfort, and, so far as I know or can ascertain, in all cities and large towns in Germany dogs are required to be muzzled whenever they are on the street or public place, and this regulation is enforced in cities even when the dog is led or held in leash by the owner or is harnessed for working purposes to a cart or other vehicle."[2]

In Great Britain the results from enforcing the muzzling order have been phenomenal, both in the opposition encountered by the authorities and in the successful eradication of the disease. The number of rabid dogs officially reported was: in 1887, 217; in 1888, 160; in 1889, 312. In the last-mentioned year muzzling was adopted, and the number of cases fell to 129 in 1890, 79 in 1891, and 38 in 1892; then, owing to persistent opposition, muzzling was stopped, and the effect of withdrawing this measure was at once seen in the increase of rabies. In 1893 there were 93 cases; in 1894, 248 cases, and in 1895, 672 cases. At this point, owing to public alarm, muzzling was again enforced, reducing the number of cases in 1896 to 438; in 1897 to 151; in 1898 to 17, and in 1899 to 9. As no case was discovered from November, 1899, to March, 1900, it was believed by the veterinary officer that the disease had been extinguished in Great Britain.

[1] Renault, cited by Bouley, in Rapport sur la Rage. Bul. de l'Acad. de Med., Paris, 1863, p. 725. Fleming. Rabies and Hydrophobia, p. 365.

[2] Consular Reports, June 19, 1900.

No stronger evidence in favor of muzzling could be asked for or desired than this complete success in an extensive and thickly populated country like Great Britain.

